# Reducing inequities in maternal and child health in rural Guatemala through the CBIO+ Approach of Curamericas: 8. Impact on women’s empowerment

**DOI:** 10.1186/s12939-022-01760-y

**Published:** 2023-02-28

**Authors:** Ira Stollak, Mario Valdez, William T. Story, Henry B. Perry

**Affiliations:** 1 Curamericas Global, Raleigh, North Carolina USA; 2 Curamericas/Guatemala, Calhuitz, San Sebastián Coatán, Huehuetenango, Guatemala; 3 Department of Community and Behavioral Health, University of Iowa College of Public Health, Iowa City, Iowa USA; 4 Health Systems Program, Department of International Health, Johns Hopkins University Bloomberg School of Public Health, Baltimore, Maryland USA

**Keywords:** Women’s empowerment, Maternal health, Child health, Community health, Primary health care, Community-based primary health care, Implementation research, Census-Based, Impact-Oriented Approach, Care Groups, Community Birthing Centers, Guatemala, Equity, Curamericas Global, Curamericas/Guatemala

## Abstract

**Background:**

Indigenous Maya women in the rural highlands of Guatemala have traditionally faced constraints to decision-making and participation in community affairs. Anecdotal experiences from previous Curamericas Global projects in Guatemala and Liberia have suggested that interventions using the CBIO+ Approach (which consists of implementing together the Census-Based, Impact-Oriented Approach, the Care Group Approach, and Community Birthing Centers), can be empowering and can facilitate improvements in maternal and child health. This paper, the eighth in a series of 10 papers examining the effectiveness of CBIO+ in improving the health and well-being of mothers and children in an isolated mountainous rural area of the Department of Huehuetenango, explores changes in women’s empowerment among mothers of young children associated with the Curamericas/Guatemala Maternal and Child Health Project, 2011–2015.

**Methods:**

Knowledge, practice, and coverage (KPC) surveys and focus group discussions (FGDs) were used to explore six indicators of women’s empowerment focusing on participation in health-related decision-making and participation in community meetings. KPC surveys were conducted at baseline (January 2012) and endline (June 2015) using standard stratified cluster sampling. Seventeen FGDs (9 with women, 3 with men, 2 with mothers-in-law, and 3 with health committees), approximately 120 people in all, were conducted to obtain opinions about changes in empowerment and to identify and assess qualitative factors that facilitate and/or impede women’s empowerment.

**Results:**

The KPC surveys revealed﻿ statistically significant increases in women’s active participation in community meetings. Women also reported statistically significant increases in rates of participation in health-related decision-making. Further, the findings show a dose-response effect for two of the six empowerment indicators. The qualitative findings from FGDs show that the Project accelerated progress in increasing women’s empowerment though women still face major barriers in accessing needed health care services for themselves and their children.

**Conclusion:**

The Project achieved some notable improvements in women’s decision-making autonomy and participation in community activities. These improvements often translated into making decisions to practice recommended health behaviors. Traditional cultural norms and the barriers to accessing needed health services are not easily overcome, even when empowerment strategies are effective.

## Background

Women’s empowerment is widely lauded as a key driver of social and economic development globally. Defined as the “utilization of their assets, opportunities, and agency for making purposive choices and engaging in behaviors to alter life circumstances” [1], empowerment can include protecting women and girls against violence and discrimination, providing opportunities for them to obtain education, and promoting their participation in health and economic decisions [2]. In low- and middle-income countries, there is consistent evidence of a favorable association between women’s empowerment and a wide array of maternal and child health outcomes, including antenatal care, skilled attendance at birth, contraceptive use, intimate partner violence, immunization, child nutrition, and child mortality [3–5].

In many regions of the world, men have the final say on issues related to women’s health, including family planning and reproductive health, and men’s limited knowledge about these issues prevents them from making informed decisions [6]. Further, women’s lack of financial autonomy and decision-making power in male-dominated societies limits women’s ability to bargain for their own health and the health of their children [7, 8]. This can have negative consequences for both women’s health and the health of their children. For example, in poor urban areas of India, it has been shown that a mother’s lack of control over financial resources is associated with lower odds of her children receiving vaccinations [9].

Guatemala is a Central American country that is home to over 16 million individuals consisting of two primary ethnic groups – the Indigenous Mayas and the *ladinos* (people of mixed Indigenous and Spanish descent, also called *mestizos*). Education is an important source of women’s empowerment [10]. But only 39% of Maya women in Guatemala are literate and they have a low level of empowerment﻿ [11]. Additionally, Maya women and children suffer from poor nutrition outcomes and experience some of the highest mortality rates in the Western hemisphere [12, 13].

The lack of agency among indigenous women in Guatemala is due, in part, to traditional gender norms that place women at a disadvantage. The traditional Maya culture is male-dominated and patrilocal: married women leave their homes to﻿ live with their husband and in-laws, where they have low status and are often treated as quasi-servants [14]. Men control the family finances and determine women’s mobility outside of the home, often under the threat of domestic partner violence [14]. Women generally lack education and are often illiterate [15]. They often do not speak Spanish and may lack awareness of many healthy household behaviors [15]. As a result, many Maya women suffer from low self-esteem, lack of control over their bodies, lack of decision-making autonomy, and limited social participation in the community [16]. In addition, the lives of Indigenous women living in the Guatemalan highlands are shaped by a cultural context of *machismo*, an exaggerated masculinity associated with hypersexuality and violence [17]. Guatemala is also noted for its high rate of murders of women, few of which result in convictions – a societal pattern referred to as *femicidio* (femicide) [18].

Improvements in women’s decision-making power and control over resources can have a positive impact on child health and nutrition as well as on maternal health [19]. Using the 1995 Guatemala Survey of Family Health, Glei and colleagues [20] identified a link between utilization of prenatal care and greater women’s decision-making autonomy. Other studies in Guatemala, however, have found that women’s decision-making power had a marginal effect on maternal health [21]. These mixed results in the context of rural, Indigenous women may be due, in part, to past negative experiences with the formal health system, which leads them to choose informal providers (e.g., traditional birth attendants) when they have the power to choose [22–24].

The context of severe gender inequity among Indigenous populations living in rural Guatemala underlines the need for developing new approaches that promote women’s empowerment and address some of the underlying societal norms that have reinforced the traditional dominance of males [25]. Experiences with previous Curamericas Global projects in Guatemala and Liberia using the Care Group Approach, together with experiences from many other Care Group projects implemented by other organizations in other settings [26–29], have suggested that Care Group participation﻿ is an empowering process, but no formal assessment of this hypothesis has been reported prior to the publication of Paper 7 in this series [30]. Paper 7 is a qualitative study that focuses exclusively on how Care Groups—one component of the Expanded Census-Based, Impact-Oriented (CBIO+) Approach described in this paper—led to the empowerment of the Project staff who supported Care Groups, the Care Group Volunteers, and the women who were taught by the Care Group Volunteers.

Papers 1 and 2 of this series [31, 32] describe the CBIO+ Approach, details about the Curamericas/Guatemala Maternal and Child Health Project, 2011–2015 (hereafter referred to as the Project), and the implementation research carried out alongside the Project. The overall Project and implementation research focused on the effectiveness of the CBIO+ Approach in improving the health and well-being of mothers and children in the Cuchumatanes mountains of an isolated section of the Department of Huehuetenango, an area inhabited almost exclusively by an Indigenous Maya population. Table 1 provides a brief description of the CBIO+ Approach. In addition to Papers 1, 2, 7 and the current paper, there are six additional papers in this series [43–48].

**Table 1:** CBIO+ explained

The CBIO+ Approach is an expansion of CBIO. It is composed of three components: (1)﻿ the Census-Based, Impact-Oriented (CBIO) Approach, (2) the Care Group Approach, and (3) the Community Birthing Center Approach. CBIO consists of conducting with the community a census, registering all households, identifying local epidemiological priorities and the health priorities according to the local people, developing and executing a plan to address these priorities, and assessing over time whether the health of the population has improved [33]. All of this is accomplished through partnerships with the community, collection of local data, and routine systematic home visitation guided by census registers to collect data, including vital events, and to deliver services. Further descriptions of the CBIO approach and its effectiveness are available [34–38]. The﻿ Care Group Approach is, in a sense, an extension of CBIO that involves the selection of one female Care Group Volunteer for every 10–15 households with a mother of young child. Then, 5–12 Care Group Volunteers meet with a Care Group Promoter every 2–4 weeks to learn 1–2 educational messages to share with the mothers in the catchment area for each Care Group Volunteer, either by visiting each home separately or meeting as a group. At the subsequent meeting, the Promoter teaches them a new message and the Care Group Volunteers report pregnancies, births and deaths to the Promoter [28]. Further descriptions of the Care Group Approach and its effectiveness are available [39–42]. The﻿ Community Birthing Center Approach, as developed by Curamericas/Guatemala, is a participatory approach that involves working with communities to construct, staff and operate a readily available local facility where mothers can give birth in a way that respects traditional customs and enables the traditional midwife (called a *comadrona* in the Project Area) to perform her traditional role. These centers are staffed 24/7 by auxiliary nurses with special additional training in midwifery and supervised by an experienced obstetrical graduate nurse who is based at one of the birthing centers and is available by phone to support the other birthing centers. Connected to each birthing center is an emergency transport system to provide prompt﻿ referral to a hospital should the need arise. Also associated with the birthing center is an insurance system that pregnant women and their families can contribute to during the pregnancy to offset to cost of transport if a referral is needed. Further descriptions of the Community Birthing Center Approach are available [23].	

The mixed-methods study reported in this paper examines the effectiveness of the CBIO+ Approach in promoting women’s empowerment and well-being by examining two hypotheses:

Hypothesis 1: Implementation of the CBIO+ Approach in communities of Indigenous Maya women produces a statistically significant increase from baseline to endline in (1) women’s health-related decision-making autonomy and (2) women’s participation in community health activities.

Hypothesis 2: There is a dose-response effect, such that the change in women’s empowerment in Area A (where the Project was present for a longer period of time) is greater than in Area B.

In addition to the quantitative testing of our hypotheses mentioned above, we sought to better understand the barriers and facilitators to women’s empowerment using qualitative methods, particularly as they affect the health of mothers and their children. The underlying theory of change, arising from previous experiences that Curamericas Global has had in the implementation of programs over the past four decades, is that effective women-centered participatory programming to improve maternal and child health provides women with a greater sense of agency in taking charge of their own lives, their own health, and the health of their children than had existed previously.

## Methods

To test the two hypotheses above, we employed a mixed-methods approach among a randomly selected sample of mothers of children 0- < 24 months of age. We utilized a quantitative survey instrument at baseline and endline concerning women’s participation in community health activities and their level of involvement in decision-making for key health practices. We also conducted focus group discussions (FGDs) midway during the Project’s implementation to explore women’s and men’s perspectives on the effect of the Project on women’s empowerment. Additional details about the methods for this study are available in other articles in this series [31, 32] as well as in the full report published online [49].

### Samples for the knowledge, practice and coverage (KPC) surveys

The Project was implemented in three Project municipalities. The Project Area was divided into two parts because we did not have the capacity to implement the CBIO+ interventions in the entire Project Area at the outset. There were 89 Area A communities that received the Project services for 44 months (October 2011 through May 2015) and 91 Area B communities that received services for 20 months (October 2013 through May 2015). Paper 2 in this series provides more details on the Project communities and the phased implementation approach [32]. The sample for this study included 299 mothers of children 0- < 24 months from 30 Area A communities at baseline and 300 at endline, and 300 mothers of children 0- < 24 months from 30 Area B communities both at baseline and at endline, with all mothers and clusters randomly selected from the implementation areas using standard stratified cluster sampling. We assumed a design effect of 2.0 to account for intra-cluster correlation [43].

The survey included demographic characteristics of the mothers: age, parity, number of years of formal schooling and ability to speak Spanish. There were very few statistically significant differences in these indicators from baseline to endline within both Areas, and very few statistically significant differences between the two Areas both at baseline and endline (data not shown).

### Quantitative data collection and analysis

In January 2012 (before household-level interventions began), a baseline KPC survey was administered to a sample of households in Area A and another sample in Area B. This survey included questions on six empowerment indicators described below. Details of the survey implementation, training of interviewers, quality control measures, and analysis are described in Paper 2 of this series [32]. The questionnaires are available from the corresponding author on request. In June 2015, an endline KPC survey was to distinct samples of women in Area A and Area B (which were different from the baseline samples). Prior to selection of clusters, the endline KPC sampling frame was modified to include updated population statistics for each community. This endline KPC survey included the same questions on the six empowerment indicators utilized in the baseline KPC survey. Altogether, four separate KPC surveys were carried out: two at baseline (in Area A and Area B) and two at endline (in Area A and Area B). We included six quantitative measures of women’s empowerment. The first two assessed women’s participation in community health activities and the last four assessed women’s health-related decision-making autonomy (Table 2).

**Table 2:** Quantitative measures of women’s empowerment

Participation in community health activities	Autonomy in health-related decision-making
1. Women’s participation in community meetings (percentage of mothers of children 0- < 24 months of age who reported that in the previous 3 months they had both attended and expressed their opinion at a community meeting)	1. Participation in decision-making regarding contraception (percentage of households with children 0- < 24 months of age in which either the mother alone or the mother jointly with her husband/partner had decided whether to use contraception)
2. Contact with a Care Group (percentage of mothers of children 0- < 24 months of age who reported that in the previous month they had been one of the following: a Care Group Volunteer, a participant in a Care Group meeting, or a recipient of instruction by a Care Group Volunteer)	2. Participation in decision-making regarding the location of delivery and the selection of the birth attendant (percentage of households with children 0- < 24 months of age in which either the mother alone or the mother jointly with another person had decided the location and birth attendant of her most recent delivery)
	3. Participation in decision-making regarding treatment of acute respiratory infection (ARI) (percentage of ARI episodes in children 0- < 24 months of age in which either the mother alone or the mother jointly with another person had decided to seek further care and treatment)
	4. Control over money for purchasing food for children (percentage﻿ of mothers of children 0- < 24 months of age who indicated that they did not need to ask for the money needed to buy food for their ﻿children)

Data entry was performed using Epi Info 7.1. Frequencies, proportions, confidence intervals, and *p*-values were calculated first with Excel and then confirmed with Epi Info 7. The statistical significance of differences in simple comparisons between baseline and endline values of indicators or between the intervention and comparison areas were calculated using WINPEPI version 11.65 (Brixton Health, London, UK). In addition, a difference-in-differences (DID) analysis was done comparing the differences from baseline to endline for indicators in Area A with those for Area B. The statistical significance of the DID estimate was assessed using a z-test based on the variances of its four component proportions.

### Qualitative data collection and analysis

FGDs were the chosen methodology due to their time efficiency and their ability to provide safe venues for the expression of individual opinions in the company of like-minded individuals. In January 2014 in Area A communities in each of the three municipalities served by the Project, we held three FGDs with women (one of these groups of women consisted of Care Group Volunteers (*Comunicadoras*), one with men, one with mothers-in-law (with the exception of San Miguel Acatán), and one with a Health Committee. Each municipality represented a unique Mayan language and ethnic group. In all, a total of 17 FGDs, that included approximately 120 people, were conducted. While the number of FGDs was determined by available time and resources, the saturation of themes achieved implies that this was a sufficient number. The number, type and location of the FGDs is presented in Table 3.

**Table 3:** Number, type, and location of focus group discussions (FGDs)

Municipality	Language/ethnicity	Area A community	Self-Help Groups members*	Men	Mothers-in-law	Health committees
San Sebastian Coatán	Chuj	Yalanculuz	1			
		Chenen	1			1
		Calhuitz	1	1		
		Lolbatzam			1	
San Miguel Acatán	Akateko	Poj Najap	1			
		Yucajo	1			
		Canton Santa Cecilia	1			
		Mete		1		
		Ixlahuitz				1
Santa Eulalia	Q’anjob’al	Buena Vista	1			
		Temux Chiquito	1			1
		Pena Flor	1	1		
		Sataq Na			1	
Total number of FGDs = 17			9	3	2	3

Note: The FGD in Yalanculuz consisted of Care Group Volunteers only

The communities were chosen randomly. The women were selected randomly from the rosters of Self-Help Groups, except for one group of women (from Yalanculuz) which was composed of the Care Group Volunteers from their community. The men and mothers-in-law were selected purposefully both by convenience and by “snowballing”, with women and Health Committees suggesting the men and mothers-in-law. Health Committees for the selected communities were interviewed in their entirety. The FGDs consisted of between 4 and 14 participants, with a median and mode of 7 participants. Each FGD took 60–75 minutes to conduct. The FGDs took place in January 2014 and were held in various community locations that afforded sufficient comfort, convenience, and privacy.

The FGDs were led by three different teams, one for each municipality, each consisting of three Curamericas Guatemala staff (*Educadoras*) who spoke the local Mayan dialect as their first language and who were also fluent in Spanish. The questionnaire (available from the corresponding author on request) had been prepared in Spanish and was translated by the team into the local idiom. The questions addressed the six empowerment indicators listed in Table 2 and are described further in Appendix 1. After the reading of a declaration of confidentiality, verbal informed consent was obtained from all participants. For lack of equipment and staff time to listen to and transcribe recordings, the FGDs were not recorded. Two bilingual members of the interview team listened to the discussion and took notes in Spanish. These notes were a combination of direct quotes and paraphrases. The notes were reviewed by the team shortly after the FGD ended to ensure accuracy and completeness. The hand-written notes were then transcribed into Word documents by a Curamericas staff member and then, to facilitate coding and analysis, entered into thematically organized Excel tables.

The analysis used both grounded theory and codification based on identification of specific facilitators and impediments to women’s empowerment. Substantive coding was used to identify themes and concepts, and then axial coding was used to combine them into macro-concepts/themes and to identify associative links between themes and concepts [50, 51].

## Results

### Quantitative findings

The evidence from the quantitative analysis provides support for Hypothesis 1, that the Project empowered women through an increase in their health-related decision-making autonomy and an increase in their participation in community health activities. Table 4 compares the six measurements of women’s empowerment at baseline and endline for both Areas A and B. When comparing the changes from baseline to endline in each Area, there was a statistically significant increase in the level of empowerment for eight of the 12 unique assessments: four in Area A communities and four in Area B communities. The most striking improvements in empowerment were in terms of Care Group contact during the previous month: the increase was 59.3 percentage points in Area A communities and 49.4 percentage points in the Area B communities. The next most notable increase in empowerment was related to decision-making concerning contraception, which increased by 27.8 percentage points in Area A communities and 27.3 percentage points in Area B communities. Improvements in women’s participation in community meetings were also notable, increasing by 14.3 percentage points in Area A communities and 17.3 percentage points in Area B communities. All of these differences from baseline to endline were statistically significant (*p* < 0.001).

**Table 4:** Comparison of women’s empowerment indicators at baseline and endline in Areas A and B in the Curamericas/Guatemala Maternal and Child Health Project, 2011–2015

	Hypothesis: Women’s empowerment improved from baseline to endline in Area A				Hypothesis: Women’s empowerment improved from baseline to endline in Area B				Hypothesis: Women’s empowerment improved more in Area A than in Area B	
Indicator	Baseline (January 2012), Area A (95% CI) ***n*** = 299	Endline (June 2015), Area A (95% CI) ***n*** = 300	Endline minus baseline	***p***-value, Baseline vs. Endline, Area A	Baseline (January 2012), Area B (95% CI) ***n*** = 300	Endline (June 2015), Area B (95% CI) ***n*** = 300	Endline minus baseline	***p***-value, Baseline vs. Endline, Area B	Is difference in Area A greater than in Area B?	***p***-value, difference in differences, Area A vs. Area B
Participation in decision-making regarding contraception	56.5% (49.6, 63.4)	84.3% (80.2, 88.4)	27.8%	< 0.001	55.7% (50.1, 61.3)	83.0% (78.7, 87.3)	27.3%	< 0.001	Yes (0.5 percentage points)	0.92
Participation in decision-making regarding location of delivery and selection of the birth attendant	68.2% (60.5, 75.9)	84.3% (73.6, 83.0)	16.1%	< 0.001	71.3% (66.2, 76.4)	76.0% (71.2, 80.8)	4.7%	0.114	Yes (11.4 percentage points)	0.02
Participation in decision-making regarding treatment of acute respiratory infection	72.7% (67.7, 77.7)	74.2% (63.3, 85.1)	1.5%	0.347	76.9% (72.1, 81.7)	89.7% (86.3, 93.1)	12.8%	< 0.001	No	0.07
Control of money for purchasing food for children	12.6% (8.8, 16.4)	11.7% (8.1, 15.3)	−0.9%	0.396	11.4% (8.7, 15.0)	7.3% (4.4, 10.2)	−4.1%	0.061	Yes (3.2 percentage points)	0.41
Contact with a Care Group	8.4% (5.3, 11.1)	67.7% (62.0, 72.9)	59.3%	< 0.001	10.3% (6.9, 13.7)	59.7% (53.9, 65.2)	49.4%	< 0.001	Yes (9.9 percentage points)	0.02
Women’s participation in community meetings	10.0% (6.6, 13.4)	24.3% (19.5, 29.1)	14.3%	< 0.001	10.7% (7.2, 14.2)	28.0% (22.9, 33.1)	17.3%	< 0.001	No	1.00

The results for the other three empowerment indicators were variable. The indicator regarding selection of birth location and birth attendant improved significantly in the Area A communities (by 16.1 percentage points, *p* < 0.001), but less so in Area B communities (4.7 percentage points, *p* > 0.05). The indicator concerning decision-making for ARI treatment showed minimal improvement in Area A (by 1.5 percentage points), but it did show a statistically significant improvement in Area B communities (by 12.8 percentage points, *p* < 0.001). Finally, the indicator related to control of money for purchasing food for children did not change in Area A, and it declined in Area B by 4.1 percentage points.

The findings partially support Hypothesis 2, that there would be a dose-response effect on empowerment. We hypothesized that the degree of improved empowerment would be greater in Area A communities than in Area B communities (because of their longer exposure to the Project). For two of the six empowerment indicators, the difference-in-differences analysis, as shown in Table 4, indicates a dose-response effect. Participation in decision-making about location of delivery and selection of birth attendant increased more in Area A than in Area B as did contact with a Care Group. However, there was no evidence of a dose-response effect for the other four indicators. And for one of these indicators (participation in decision-making regarding treatment of acute respiratory infection), the improvement was greater in Area B than in Area A (*p* = 0.07).

The quantitative data also provide some insight into the facilitators and barriers to women’s empowerment. As shown in Table 4, the overall percentage of mothers who had control of money for purchasing food for children remains consistently low (13% or less) and showed no improvement over the course of Project implementation. Participation in community meetings, though it improved in both Areas A and B from around 10% to 24–28%, the level at endline remained low, particularly when compared to the remaining four empowerment indicators at endline (all having a level of 60% or higher).

### Qualitative findings

Most FGD participants of all informant types (reproductive age women, husbands/partners of women of reproductive age, mothers-in-law of reproductive age women, and members of Community Health Committees) noted improvements in the capacity of women to control and direct their own lives. Each of the 17 FGDs generally agreed that the work of Curamericas/Guatemala had improved the situation for women’s empowerment. The most frequently cited reasons included: the health education provided by the Care Group Volunteers (*Comunicadoras*); the improved capacity of mothers to take care of their children﻿, the lessening of women’s timidity and their fear of participating in community meetings, and the opportunity to meet monthly and talk among themselves (Table 5).

**Table 5:** Responses of focus group participants to query about how the Curamericas/Guatemala Project empowered women

Category of response
Through the monthly health education provided by Care Group Volunteers
Through learning how to take better care of their children (providing better nutrition, including exclusive breastfeeding, better hygiene, and better care of their children when they are sick)
By helping mothers lose their fear of expressing themselves in front of others and learning to make their voices heard
Through the opportunity to meet monthly, talk, participate, and share opinions
Through their access to basic health services
Through the education provided at the Birthing Centers (*Casas Maternas Rurales*)
Through visits to the home by Promoters and Care Group Volunteers
By being able to contribute their ideas and having these ideas given consideration

Representative comments from the women’s FGDs regarding the Project’s effect on women’s empowerment included the following:

*They* [the Project] *give us the opportunity to speak and participate and express our opinions.*

-Women’s FGD participant

*All of us now know our rights and obligations.*

-Women’s FGD participant

*We go to the trainings where we receive education on health and nutrition. This has ﻿helped us because we practice and see the change. Our children don’t get sick* [as often]. *We wash our hands, which wasn’t so important* [before]. *But now we try to change our behavior.*

-Women’s FGD participant

*Now we aren’t afraid to participate [in community activities]*

-Women’s FGD participant

*We bring ideas and they are considered by others.*

-Women’s FGD participant

*It is important for us to value our rights and to participate and take on formal posts in the community.*

-Women’s FGD participant

*Before women had no rights, but this has changed and we now have our rights.*

-Women’s FGD participant

*Yes, because we are owners [dueñas] of our lives and no one can obligate us to do anything we don’t want to do.*

-Women’s FGD participant

*Now men give more freedom to women and mistreat women less.*

-Women’s FGD participant

The men’s FGDs also all agreed that Curamericas/Guatemala had facilitated a change in empowerment of women through the following: the Community Birthing Centers (called﻿ *Casas Maternas Rurales* and referred to locally as *Casas* and described further in Papers 1 and 6 [31, 46]), the education and health services provided to the women, the bringing of women together and the encouragement of them to speak and participate, and the general community development fostered by the Project.

Representative comments include the following:

*Curamericas has helped this change through the education that it provides to women.* -Men’s FGD participant.

*Curamericas helped facilitate the change through the Casas.* -Men’s FGD participant

*Yes, because before* [the women] *didn’t have the knowledge of how to take care of their children, but nowadays they are well trained and now they participate* [in community affairs]. -Men’s participant

*Women now don’t have the fear that they have had.* -Men’s participant

*Nowadays women are very well trained to execute well their own activities and projects.* -Men’s participant

The two FGDs composed of mothers-in-law of mothers of young children agreed that Curamericas/Guatemala had facilitated a change in empowerment by providing staff who came to educate women about how to care for themselves and their children; providing counsel about exclusive breastfeeding; providing medicines; improving practices of nutrition, hygiene, and care seeking for sick children; and raising community consciousness about the importance of health care. Several﻿ representative quotes are as follows:

*Everyone says now that mothers now know how to provide good hygiene and nutrition in the home and take good care of their children when they are sick.* -Mother-in-law FGD participant

*Women are now supported in going to the Self-Help Group meetings, something that was prohibited to them before,* -Mother-in-law FGD participant

*Women can make decisions about their lives now – they have rights, there is more cleanliness, and they have knowledge about feeding and hygiene.* -Mother-in-law FGD participant

Participants from all three Community Health Committees concurred that Curamericas/Guatemala had facilitated changes in women’s empowerment by means of the health education talks, home visits, the advice provided by the birthing center staff, teaching about the very sensitive subject of family planning, and, in general, the Project's overall support for women and their children. They noted improved health practices at the family level; greater participation of women, whose voices were now heard; and more women in positions of leadership due to Curamericas/Guatemala’s efforts. Community Health Committee FGD participants mentioned the following:

*Yes, because now there are women heads* [of communities]﻿ *and women facilitators of community work.* -Community Health Committee FGD participant

*Yes, in most part because the women participate more and now make their voice heard.* -Community Health Committee FGD participant

*In a recent community meeting the majority of those attending were women, and their opinions and decisions were respected.* -Community Health Committee FGD participant

*Women’s participation in this last year has been very active, and now they participate more and express their ideas.* -Community Health Committee FGD participant

*Women are now making their own decisions thanks to the various programs that are working with them.* -Community Health Committee FGD participant

#### Additional findings related to facilitators of and barriers to women’s empowerment

In addition to uncovering how the Project contributed to women’s empowerment, the qualitative study was designed to elicit facilitators and barriers to women’s empowerment in the Project Area so that the Project could possibly influence those factors to increase empowerment going forward. Across the FGDs the FGDs, multiple themes emerge about what facilitates and what impedes women’s empowerment. The detailed findings from the FGDs regarding these issues as expressed by both men and women who participated are available in the full report of the implementation research [52]. Table 6 summarizes these findings. Additional qualitative findings that support those provided here are available elsewhere [49].

**Table 6:** Facilitators and impediments to women’s empowerment expressed in FGDs with both men’s and women’s groups

Facilitators of empowerment	Impediments to empowerment
**Level of self-esteem and self-confidence**	
High﻿ self-esteem and self-confidence Little or no fear of expressing oneself in the presence of men or of assuming community ﻿responsibilities	Low self-esteem Timidity Fear of speaking in presence of men Fear of ridicule Fear of failure Reluctance to assume community responsibilities Fear of taking advantage of opportunities to participate in community meetings
**Level of education/Spanish fluency**	
Education﻿ (both formal and informal) Fluency in Spanish	Lack of education Limited fluency in Spanish
**Level of consciousness of rights and self-ownership**	
Awareness﻿ of women’s own civil and human rights	Lack of awareness of women’s civil and human rights
Sense﻿ of being owner (*dueña*) of one’s own body	No sense of being owner (*dueña*) of one’s own body; husband is owner (*dueño*) of woman and family
**Degree to which relationship with husband/family is supportive**	
General﻿ support from husband (and to a lesser extent from the mother-in-law and/or the woman’s parents)	Domination by husband (and to lesser extent, mother-in- law and/or woman’s parents)
Trust﻿ from husband (that his wife will comport herself well, remain faithful, handle ﻿money and responsibilities well, and make sound decisions)	Lack of trust from husband (that his wife will comport herself well, remain faithful, handle money and responsibilities well, and make sound decisions)
Good﻿ communication with the husband, ability to negotiate her mobility and participation﻿ in decisions	Poor or no communication with husband; inability to negotiate her mobility and her participation in decisions
﻿Mobility (ability to leave the household, especially alone, to participate in meetings and ﻿community activities, with or without husband’s permission)	Lack of mobility (unable to leave home; forbidden or requires husband’s explicit permission, or required to be accompanied by others)
Permission﻿ of husband to participate is not needed or easily granted – often only as a formality or just to know the woman’s whereabouts	Permission of husband to participate is not given, or given grudgingly or conditionally (e.g., after household chores are done)
No fear of husband’s anger or of intra-familial violence	Living in fear of angering husband/provoking “problems” such as domestic violence
Ability to participate (at least nominally) in most decisions regarding place of delivery, family planning, and care seeking for sick children Recognition by family that “mother knows best” regarding place of delivery or care seeking for sick children	Being ignored or over-ruled by husband and/or mother-in-law in health-related decision-making
Absence of husband – out of town or away working as migrant laborer	Presence of [unsupportive] husband living in household
**Level of control over management of household responsibilities**	
Ability to balance role as participant in community meetings/activities with traditional role as housewife/mother Ability to not let household responsibilities impede participation in community meetings/activities	Feeling too burdened by household and childcare responsibilities to leave the home to participate in community meetings/activities Acceding to the traditional housekeeping/childcare role that keeps women isolated in the home
**Level of economic autonomy**	
Produces her own income that she controls (or she has some control over her husband’s/partner’s income)	Economic dependence on husband Traditional role of husband as breadwinner (the money he earns is “his”, with no sense of joint ownership) Woman does not generate her own income/money that is “hers”

## Discussion

Our findings provide evidence to support the hypothesis that the Curamericas/Guatemala Maternal and Child Health Project, 2011–2015, facilitated increased empowerment of mothers of young children in the Project Area – according to (1) reports of a representative sample of mothers participating in household surveys about various aspects of their agency related to participation in community activities and in health-related decision-making as well as (2) the opinions of mothers, husbands/partners, mothers-in-law, and members of Community Health Committees participating in FGDs. In this male-dominated context, the increase in female participation in community affairs and in health-related decision-making within the family is an important achievement. The high level of participation of mothers in the Care Group process at endline (60–68%) indicates that the majority of mothers were exposed to a participatory and empowering group activity. There were also notable improvements in mothers’ engagement in decision-making processes from baseline to endline, especially those related to contraception (a difference of 27.8 percentage points between baseline and endline in the Area A communities and a difference of 27.3 percentage points in the Area B communities). Given the evidence documented elsewhere in this series [31, 43, 44, 47] of the extensive activities carried out by the Project that involve mothers and the community more broadly and the absence of any other activities that might have produced these findings, it is reasonable to conclude that the Project was responsible for at least some of the improvements﻿ shown here.

The second hypothesis, that there would be a dose-response effect showing more change in empowerment in Area A than in Area B because of the longer time of Project implementation there was only partially confirmed – by two of six quantitative indicators of empowerment. Part of the reason for this appears to be the quick uptake of the “dose” in Area B, as seen as well for other indicators besides women’s empowerment.

The lack of progress in control over money for purchasing food for children in both Areas A and B is notable, perhaps reflecting the persistent influence of male dominance over this critical aspect of family decision-making. The lack of progress in Area A in participation in decision-making regarding treatment of acute respiratory infection in comparison to Area B, where significant progress was made, has no obvious explanation. One conjecture is that the field staff had matured and become more effective at the time the work in Area B began, and this could have accelerated progress in helping women in Area B seek care for their child when symptoms of acute respiratory infection arose.

Issues of empowerment of women and health in low-income countries have been frequently addressed in the peer-reviewed literature. However, in general, these publications have focused on the positive association between empowerment and health [53, 54] rather than on the empowering effects of participating in community health programs. The empowering effect of participatory community-based primary health care programs (including participation in women’s self-help groups) has been less well studied and results have been mixed.

One quantitative study from rural Nepal [55] assessed whether women’s Participatory Learning and Action Groups (that had been effective in reducing maternal and neonatal mortality) had an effect on women’s agency. No impact was identified. A qualitative study from rural Uganda assessed the impact of Participatory Learning and Action Groups on women’s empowerment and reported favorable changes in communication skills, networking, self-confidence, and an increase in their social status [56]. Kumar et al. [57] carried out a large-scale quasi-controlled assessment of the empowerment effects of women’s self-help groups in India that were originally established as savings and credit groups but were expanded to focus on health and nutrition, improving governance, and addressing social issues related to gender- and caste-based discrimination. Improvements were noted in the aggregate score on women’s empowerment, but there were no statistically significant changes in attitudes towards domestic violence and respect within the household.

The general pattern observed from the quantitative findings of our study was an improvement in women’s power to control and direct their own lives, but still in a context of traditional male domination that represents a stubborn impediment to women’s empowerment. This is a common occurrence in societies undergoing a gender transition, where early in the transition greater women’s empowerment challenges hegemonic masculine norms and can result in men’s restrictive behavior, including intimate partner violence. As women’s empowerment becomes normative over time to accommodate a more gender-equitable society, a reduction in gender-based violence and better health outcomes for women takes place [58, 59]. Based on the understanding obtained through this study that the husband’s control of his wife’s mobility affected her participation in Care Groups and community meetings, in March 2014 the Project started targeting husbands with behavior change communication to alleviate this barrier. Male Health Educators were also added because their messaging was more readily accepted by other men.

The FGDs provided rich insights from community members regarding the various facilitators and impediments to women’s empowerment. The CBIO+ Approach provided opportunities for women to come together in Care Groups and Self-Help Groups, which contributed to greater self-confidence, more equitable relationships within the household, and greater decision-making autonomy as reported by the FGD participants. The qualitative findings helped to explain how some of the quantitative empowerment effects observed in this study were attained. The facilitators and impediments outlined by the FGDs also point to the multi-dimensionality of women’s empowerment and the complex processes involved in improving the empowerment of women in the traditional, rural Maya context. Nonetheless, the changes which the Project facilitated are noteworthy.

Our quantitative and qualitative findings reported here, along with improvements in key maternal and child health indicators as shown in the third paper in this series [43], suggest that the area in which women appear to be most consistently empowered is with respect to health-related decision-making – namely, participation in decision-making regarding family planning, location of delivery and choice of birth attendant, and seeking care for a sick child. We recognize that the form of empowerment that we are describing here is one in which women are, in a sense, passive recipients of opportunities that are being presented to them by the Project for their benefit rather than serving as agents of their own change. Nonetheless, at least in issues related to maternal and child health, support provided by those with technical and professional skills are necessary adjuncts to this process.

Within the family context, it is clear that women’s empowerment must accompany a change in the man’s traditional role of *jefe* (“chief”/“boss”) and *dueño* (“owner/manager”) of his spouse, and an increasing sense on her part that she is *dueña* of her own body and entitled to the accompanying rights and responsibilities. A common theme that emerged from the FGDs, including those composed of men, was that the husband/partner is the gatekeeper – the key facilitator or impediment to female empowerment. In most cases a woman’s capacity to participate fully in community affairs is not something intrinsically hers, but rather something granted by those controlling her life – generally her husband/partner. This repressive domestic environment instills in women low self-esteem, fear of failure, feelings of timidity and shame, and lack of interest in affairs outside the home, cited by many women as impediments to their empowerment. Men’s affirmation of their partner’s agency is associated with better women’s health and well-being outcomes as well as better access to care for women and children [60]. It may take time to observe the full transformational impact of the Project on empowerment of women in the Project Area.

### Strengths and limitations

This study combines qualitative and quantitative methods to demonstrate that the Project made notable progress in improving the empowerment of women in the Project Area. The utilization of 17 FGDs with various types of respondents, including men, and a total of approximately 120 participants that were broadly representative of the Project Area is a strength.

In spite of its many strengths, there are nonetheless some limitations that should be kept in mind. First, the FGDs were not conducted at the end of the Project, but rather at a mid-Project assessment, 1.5 years before the Project ended. It is quite likely that the qualitative findings might have been even more convincing regarding the Project’s influence on women’s empowerment if they had been collected at the end of the Project. Second, the men’s groups and the mothers-in-law groups may not have been representative of their category of informants. However, even though these respondents were not randomly selected, the communities from which they came were randomly selected. Third, cultural and language barriers could have hampered communication, leading to some lost information. The note-takers, for instance, who were fluent in the Maya language being spoken, listened to the FGDs conducted in the local Maya language but took notes in Spanish, which may have risked mistranslation or loss of subtleties of meaning. Fourth, the FGD questions explicitly asked what or who facilitated or impeded specific behavioral indicators of empowerment. Thus, the questions structured the responses and therefore﻿ directly influenced the coding. This may have inhibited freer discussion among the participants. Finally, the fact that Project staff members (Health Educators) led the FGDs could have the FGD participants’ responses to be more favorable toward the Project and its impact than might have been the case otherwise. Nonetheless, we think that the main messages from the respondents did get through and they represent the views of men and women in the Project Area.

The quantitative data have some limitations that merit mention as well. The measurement of a complex construct such women’s empowerment is a challenge. The six questions that we included in the survey of mothers to measure empowerment was our best effort to obtain a quantitative measure of women’s empowerment. Some of these questions had been used in other studies that assessed women’s empowerment [61]. In addition, we must recognize that social desirability bias may have been present, meaning the respondents may have had a predisposition to respond to questions in a way that they think the interviewer “wanted” to hear. However, at least for the KPC survey questions, this bias would presumably have been similar at baseline and endline and therefore have had a limited effect on any differences observed.

## Conclusion

We observed statistically significant increases in women’s empowerment as determined from household surveys with mothers as well as strong confirmation of increases in women’s empowerment cited during FGDs with mothers, husbands/partners, mothers-in-laws, and Community Health Committee members. These findings are consistent with the assertion that the CBIO+ Approach as implemented by Curamericas/Guatemala in the Western Highlands of Guatemala is empowering to women participants in the Project. Although we cannot with certainty exclude the possibility that some extraneous influence or set of influences produced these results, such an effect seems highly unlikely in light of the numerous community-based activities with women that the Project engaged in. The empowerment gained will be difficult to translate into improvements in health-related actions unless the knowledge or the material resources needed to make and execute better decisions are available. This means that activities that promote women’s empowerment must be accompanied by the provision of accessible services that enable women to actualize that empowerment, such as available and affordable transportation, available and affordable user-friendly and properly-stocked clinics, and more locally available Community Birthing Centers*.*

## Data Availability

All of the Project reports, de-identified data, as well as publications about the Expanded CBIO+ Approach cited in this article are available from the corresponding author on request.

## Appendix 1

### Questions asked of women in focus group discussions

**These are approximate translations from Spanish:**

1. Do you think that in your community women have the power to control and lead their own lives? What limits or facilitates women’s power to control and lead their own lives?
2. Did you attend recent community meetings and express your opinions or ideas? What limited or aided you in doing this?
3. Did you participate in any Self-Help Groups [led by Care Group Volunteers]? What limited or aided your participation in these?
4. Are you using a method of family planning? Did you decide what method to use? Did anyone else participate in that decision?
5. When you were last pregnant, who decided where you would give birth?
6. When you﻿r child was last sick with symptoms of acute respiratory infection, was treatment sought? Did you participate in that decision?
7. When it is necessary to purchase medicines, health care, or food for your child, do you have to seek permission from your husband/partner for this?
8. Is the situation of women and their ability to control their own lives changing in your community? In what way? Has the Curamericas/ Guatemala Project for mothers and children aided this in any way? If so, how?

## Abbreviations

ARI: Acute respiratory infection
CBIO: Census-Based, Impact-Oriented Approach
CBIO+: Combination of the CBIO Approach with Care Groups and *Casas Maternas Rurales*
FGD: Focus group discussion
KPC: Knowledge, practice, and coverage (household survey)
